# Severe pneumonia caused by *Nocardia otitidiscaviarum* in a patient with bronchiectasis and IgA nephropathy: a case report

**DOI:** 10.3389/fmed.2025.1496814

**Published:** 2025-02-04

**Authors:** Yi Lin, Zhao-Zhao Jiang, Xiao-Qian Chi, Jian-Sheng Chen, Chao Wen, Chao Zhang, Ying-Ying Wang, Guang-Liang Xie

**Affiliations:** ^1^Department of Nephrology, Ruian Hospital of Traditional Chinese Medicine, Wenzhou, Zhejiang, China; ^2^Department of Clinical Laboratory, Ruian Hospital of Traditional Chinese Medicine, Wenzhou, Zhejiang, China; ^3^Department of Nephrology, Yueyang Hospital of Integrated Traditional Chinese Medicine and Western Medicine, Shanghai University of Traditional Chinese Medicine, Shanghai, China

**Keywords:** pneumonia, *Nocardia otitidiscaviarum*, bronchiectasis, case report, linezolid, trimethoprim-sulfamethoxazole, hormone, IgA nephropathy

## Abstract

**Background:**

*Nocardia* species are rare opportunistic pathogens in the clinic, with strong invasiveness and dissemination, that can cause serious pulmonary infection, especially in immunocompromised patients, chronic lung diseases and hormone use, and is easy to be missed and misdiagnosed, preventing patients from obtaining timely and effective treatment, resulting in a high mortality rate.

**Case presentation:**

Here, we present a rare case of a patient with chronic bronchiectasis and IgA nephropathy who developed *Nocardia otitidiscaviarum* pneumonia shortly after hormone therapy. The patient presented with tongue and lip ulcers, chest distress, cough, expectoration, and fever as the initial symptoms, which were extremely similar to common bacterial pulmonary infections. The laboratory examination and pulmonary computer tomography results indicated pulmonary infection, but the blood and multiple sputum cultures failed to identify the pathogen. Empirical treatment with piperacillin/tazobactam sodium and ceftriaxone was ineffective, and the patient’s condition worsened and progressed to respiratory failure. Subsequently, a bronchoscopy examination was performed, and the bronchoalveolar lavage fluid was collected for bacterial culture, which indicated *Nocardia* infection, however the treatment used of trimethoprim-sulfamethoxazole combined with imipenem was not effective. Finally, the patient was confirmed to have *Nocardia otitidiscaviarum* infection by mass spectrometry. According to the antibiotic sensitivity test and minimum inhibitory concentration (MIC) value results, *Nocardia otitidiscaviarum* was resistant to imipenem, so the treatment was changed to trimethoprim-sulfamethoxazole combined with linzolid. The patient’s condition improved rapidly and he was discharged after his condition was stable.

**Conclusion:**

This case reminded us that for patients with a history of chronic lung disease, when pulmonary infection occurs during hormone or immunosuppressive therapy for kidney disease, the possibility of *Nocardia* infection should be fully considered, and high-quality specimens should be collected as early as possible. Appropriate bacterial culture methods and efficient identification techniques should be adopted to promptly identify pathogens, and personalized treatment plans should be developed based on antibiotic sensitivity tests to save patients’ lives.

## Introduction

*Nocardia* species are widely present in natural environments, especially in soils rich in organic matter, decaying vegetation, and stagnant water, Gram staining and weak acid-fast staining are both positive (1, 2). They are conditional pathogenic bacteria, first discovered by Edmond Nocard in 1888. Currently, 119 species have been identified, of which 54 can cause human infections (2). *Nocardia* related infections occur sporadically all over the world. The annual incidence rate in North America, Europe and Australia is about 0.375 cases/100000, which is related to work and environmental exposure, and about 60% of them have immune dysfunction. In addition, hormones and immune preparations are also considered risk factors for *Nocardia* infection. Despite the increasing number of reports related to *Nocardia* in recent years, there is still no large-scale epidemiological data or prospective studies, and there is still a lack of consensus on the best empirical treatment. The clinical symptoms of *Nocardia* pneumonia are non-specific, and some types of *Nocardia* can easily lead to disseminated infections. Moreover, *Nocardia* ususally grows slowly and is prone to missed diagnosis, resulting in a high mortality rate for patients who do not receive timely and effective treatment. Compared with other *Nocardia*, infections caused by *Nocardia otitidiscaviarum* (*N. otitidiscaviarum*) are relatively rare, accounting for only 3% to 5% of all reported *Nocardia* infections (3, 4). Herein, we report a case of a patient with chronic bronchiectasis and IgA nephropathy who developed *N. otitidiscaviarum* pneumonia shortly after hormone therapy.

## Case presentation

**FIGURE 1:**
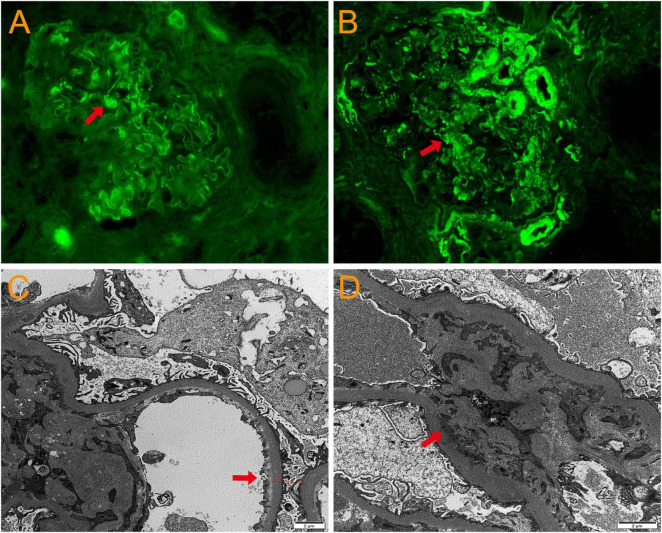
Pathological results of renal tissue biopsy. Immunofluorescence: immunoglobulin IgA **(A)** and complement C3 **(B)** deposited in clumps and granules in the mesangial area and capillary loops of the glomerulus. Original magnification, × 400. **(C)** Electron microscopy: thickening of the basement membrane of glomerular capillary loops. Original magnification, × 3000. **(D)** Electron microscopy: electron dense deposition in the glomerular mesangial area. Original magnification, × 3000.

**FIGURE 2:**
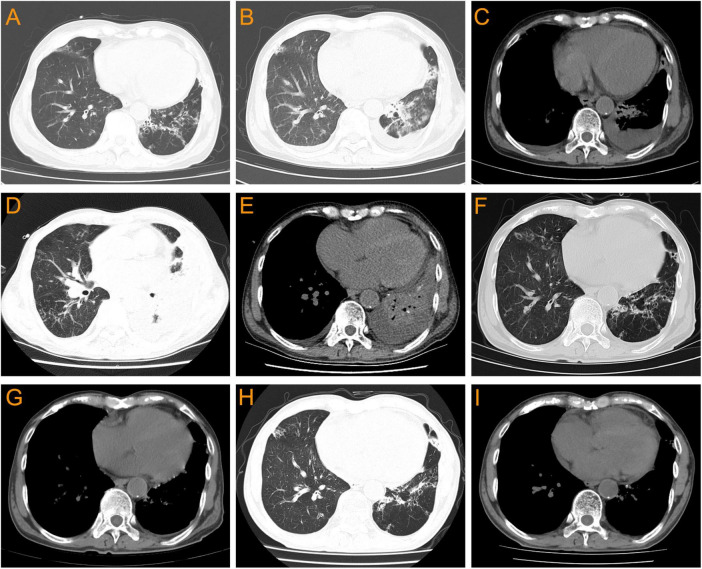
Pulmonary CT changes of this patient before and after *Nocardia* infection. **(A)** 12 days before admission (with no *Nocardia* infection): bronchiectasis without obvious pulmonary infection or pleural effusion. **(B,C)** On admission: bilateral bronchiectasis with pulmonary infection and bilateral pleural effusion. **(D,E)** On the 14th day of admission: worsening of bilateral pulmonary infections and bilateral pleural effusion, accompanied by left atelectasis. **(F,G)** 12 days after linezolid combined with TMP-SMZ treatment: bilateral pulmonary infection improved significantly, with a small amount of left pleural effusion. **(H,I)** 2 months after discharge: bronchiectasis with no obvious pulmonary infection and pleural effusion.

A 69 year-old man was admitted to the hospital due to “cough, expectoration, and fever for 2 days”. He had a history of hypertension for 10 years, chronic bronchiectasis for 10 years, and underwent surgical treatment for bladder and prostate tumors 2 years previously. 4 years ago, he was found to have an increase in serum creatinine (Scr) with a maximum of 120 μmol/L. Since then, he had been irregularly followed up with Scr fluctuating between 110 and 130 μmol/L. 2 months before admission, due to unexplained proteinuria and rapid increase in Scr (up to 294 μmol/L), a renal tissue biopsy was performed. Pathological results showed that immunoglobulin IgA and complement C3 were deposited in clumps and granules in the mesangial area and capillary loops of the glomerulus (Figures 1A–D). The patient was then diagnosed with “IgA nephropathy,” but he refused treatment. 12 days before admission, after pulmonary computer tomography (CT) examination to rule out pulmonary infection and other potential infections (Figure 2A), the patient was treated with prednisone 30 mg/day orally due to increased proteinuria and progressive lower limb edema. At the same time, trimethoprim-sulfamethoxazole (TMP-SMZ) was given 0.24 g/day to prevent potential infection. 4 days before admission, the patient developed tongue and lip ulcers for unknown reasons. 2 days before admission, the patient began to experience mild chest distress, occasional cough and purulent sputum, without chest pain or fever. The patient’s laboratory results are displayed in Table 1.

**TABLE 1:** Laboratory results of the patient during hospitalization.

Date	On admission	14 days after hospitalization	3 days after imipenem treatment	12 days after linezolid treatment	Reference range
WBC	8.3 × 10^9^/L	10.56 × 10^9^/L	11.03 × 10^9^/L	4.01 × 10^9^/L	3.5–9.5 × 10^9^/L
Neutrophils	96.3 %	82.9 %	80.3%	74%	40–75%
Hemoglobin	94 g/L	93 g/L	97 g/L	99 g/L	130–175 g/L
Blood platelet	161 × 10^9^/L	97 × 10^9^/L	104 × 10^9^/L	178 × 10^9^/L	125–350 × 10^9^/L
Lymphocyte	2.1 %	9.7 %	11.2 %	18 %	20–50%
C-reactive protein	0.79 mg/L	148.76 mg/L	178.5 mg/L	6.4 mg/L	<10 mg/L
Procalcitonin	0.12 ng/L	0.54 ng/L	0.4 ng/L	0.35 ng/L	<0.5 ng/L
Alanine transaminase	10 U/L	10 U/L	15 U/L	16 U/L	9–50 U/L
Aspartate aminotransferase	15 U/L	20 U/L	27 U/L	23 U/L	15–40 U/L
Total protein	51.1 g/L	44.7 g/L	46.9 g/L	57.8 g/L	65–85 g/L
Albumin	28.6 g/L	20.9 g/L	25.3 g/L	30.7 g/L	40–55 g/L
Serum creatinine	304 μmol/L	355 μmol/L	333 μmol/L	316 μmol/L	40–110 μmol/L
24-h urine protein	6.01 g/24 h	4.78 g/24 h	3.62 g/24 h	4.33 g/24 h	<0.15 g/24 h

On the day of admission, the patient went to the hospital due to worsening chest distress and cough, expectoration, and fever. The laboratory examination results showed that WBC 8.3 × 10^9^/L, neutrophils 96.3%, lymphocyte 2.1%,C-reactive protein (CRP) 0.79 mg/L, and procalcitonin 0.12 ng/L. The body temperature was 38.7^0^C, and the arterial oxygen saturation was normal. Pulmonary CT results showed bronchiectasis with scattered infections, emphysema, and a small amount of bilateral pleural effusion (Figures 2B, C). The patient was then diagnosed with pneumonia. After collecting blood and sputum samples for bacterial culture, empirical treatment with piperacillin/tazobactam sodium (9.0 g/day) was administered. However, the patient’s clinical symptoms did not show significant improvement and there was still recurrent fever. During the treatment period, a total of one blood culture, four sputum cultures, as well as fungal (1, 3)-β-D glucan detection, fungal galactomannan antigen detection, and tuberculosis infection T cell spot test were performed, but the results were all negative.

On the 14th day of admission, the patient experienced a progressive decrease in blood oxygen saturation, with a minimum of 85%. Pulmonary CT results showed worsening of bilateral pulmonary infections and pleural effusion, accompanied by left atelectasis (Figures 2D, E). The laboratory examination results showed that WBC 10.56 × 10^9^/L, neutrophils 82.9%, lymphocyte 9.7%, CRP 148.76 mg/L, and procalcitonin 0.54 ng/L. Subsequently, bronchoscopy was performed to collect bronchoalveolar lavage fluid (BALF) for laboratory examination and bacterial culture. The BALF was grayish-white, with a nucleated cell count of 2120/μL, while a red blood cell count of 3840/μL, neutrophils 89%, lymphocytes 4%, and alveolar phagocytic cells 7%. The BALF smear examination only detected oral and pharyngeal microbiota, with no fungi or *Mycobacterium tuberculosis*, while fluorescence staining also did not detect acid-fast bacilli. Besides, *Aspergillus* antigen was negative and fungal (1, 3)-β-D glucan <37.5 pg/mL. Then piperacillin/sulbactam sodium was discontinued and the antibiotic was changed to TMP-SMZ (5.76 g/day) combined with ceftriaxone (2.0 g/day).

**FIGURE 3:**
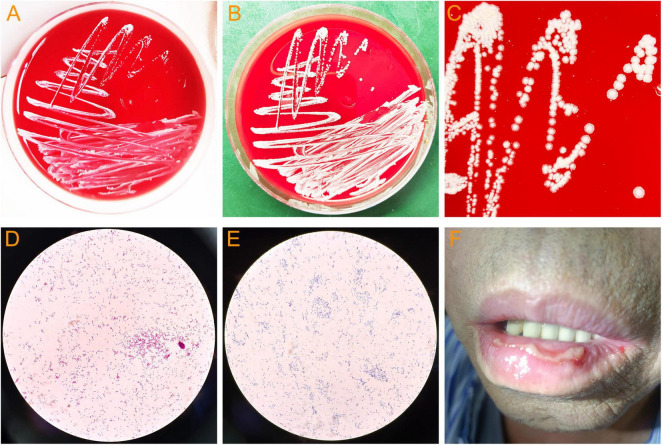
Bacterial culture of bronchoalveolar lavage fluid and lip ulcers in patient. **(A)** Colonies of *N. otitidiscaviarum* cultured for 2 days. **(B)** Colonies of *N. otitidiscaviarum* cultured for 5 days. **(C)** Colony morphology of *N. otitidiscaviarum*. **(D)** *N. otitidiscaviarum* by Gram staining. **(E)** *N. otitidiscaviarum* by weak acid-fast staining. **(F)** Lip ulcers in patient (This image was obtained with the patient’s informed consent).

In addition, The BALF culture results showed that pin-sized white colonies could be seen after 2 days, but it was difficult to identify the bacteria species (Figure 3A). Large, dry, white colonies were observed after 5 days (Figures 3B, C), which were Gram staining positive and weak acid-fast staining positive (Figures 3D, E), indicating *Nocardia* infection, a bacterium widely present in soil and dust. Upon further inquiry, it was found that the patient enjoyed planting flowers, indicating a risk of close contact with *Nocardia* in the soil, so the patient was considered to be infected with *Nocardia*. According to the US guidelines (5), imipenem was the preferred empirical treatment for *Nocardia*, so ceftriaxone was discontinued on day 19 of admission and imipenem (2.0 g/d) was used instead. However, the patient’s condition did not improve after 3 days of imipenem combined with TMP-SMZ treatment, and chest tightness, fever, and respiratory failure were still not relieved. Besides, the patient experienced mild headaches, listlessness, and slow reaction that lasted for several days. A head magnetic resonance imaging (MRI) examination was performed, and the results showed white matter lesions (Fezekas grade 2) without any signs of brain abscess. Finally, the cultured bacteria was confirmed to be rare *N. otitidiscaviarum* by VITEK MS (bioMérieux, France) with a confidence value of 99.9%, and as shown in Table 2, the antibiotic sensitivity test results and minimum inhibitory concentration (MIC) value indicated that it was resistant to imipenem, but sensitive to TMP-SMZ and linezolid. Linezolid was also one of the recommended drugs for the treatment of *Nocardia* infection (5). Due to the lack of improvement in the patient’s condition after receiving imipenem treatment, and based on the results of drug sensitivity tests, imipenem was discontinued and replaced with linezolid (1.2 g/day). After 3 days of treatment with linezolid and TMP-SMZ, the patient’s symptoms of chest distress, cough, expectoration and fever were significantly improved, the tongue tip ulcer disappeared, and the lip skin ulcer gradually improved (Figure 3F). The patient’s arterial oxygen saturation increased to over 98%, and the above-mentioned central nervous system symptoms disappeared. Afterwards, the patient’s condition continued to improve. After 12 days of linezolid combined with TMP-SMZ treatment, pulmonary CT showed a significant improvement in pulmonary infection compared to before, only with a small amount of pleural effusion on the left side (Figures 2F, G), and the laboratory examination results showed WBC 4.01 × 10^9^/L, neutrophils 74%,lymphocyte 18%,CRP 6.4 mg/L, and procalcitonin 0.35 ng/L. After 20 days of treatment with linezolid, the patient’s respiratory symptoms and lip ulcers disappeared. In the course of treatment, considering the patient’s renal dysfunction and the toxic side effects of TMP-SMZ on the kidneys, we gradually reduced the dosage of TMP-SMZ until discontinuation. Besides, the dose of prednisone was also gradually reduced from 30 mg/day to 5 mg/day during hospitalization.

**TABLE 2:** Susceptibility of *Nocardia otitidiscaviarum* isolate to different antimicrobials.

Antimicrobials	MIC	Unit	Susceptibility	Breakpoints
Trimethoprim-sulfamethoxazole	0.5	μg/mL	S	≦2, ≥4
Cinocycline	0.5	μg/mL	S	≦1, ≥8
Ciprofloxacin	4	μg/mL	R	≦1, ≥4
Levofloxacin	8	μg/mL	R	≦1, ≥4
Clarithromycin	>16	μg/mL	R	≦2, ≥8
Linezolid	1	μg/mL	S	≦8, –
Tobramycin	≦1	μg/mL	S	≦4, ≥16
Amikacin	≦4	μg/mL	S	≦8, ≥16
Imipenem	>32	μg/mL	R	≦4, ≥16
Cefatriaxone	>128	μg/mL	R	≦8, ≥64
Amoxicillin/clavulanic acid	>32	μg/mL	R	≦8, ≥32

MIC, minimum inhibitory concentration; S, susceptible; I, intermediate; R, resistant. Susceptibility of the isolate to antimicrobials was defined according to the CLSI M24-A guidelines.

**FIGURE 4:**
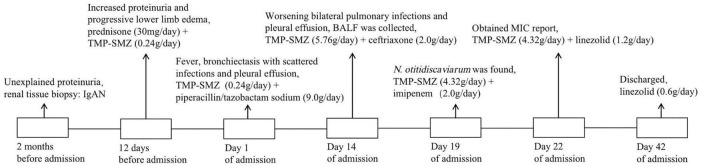
Timeline of key events. IgAN, IgA nephropathy; TMP-SMZ, trimethoprim-sulfamethoxazole; BALF, bronchoalveolar lavage fluid; MIC, minimum inhibitory concentration.

After 42 days of hospitalization, the patient was discharged and continued to receive long-term oral treatment with linezolid at a dose of 0.6 g/day (The timeline of key events is shown in Figure 4). After a 2-month follow-up after discharge, except for occasional cough, the patient had no chest tightness, sputum or fever, and pulmonary CT showed no obvious infection or pleural effusion (Figures 2H, I).

## Discussion

*Nocardia* infection tends to occur in patients with weakened immune function, altered lung structure, and chronic lung disease. *Nocardia* can cause local or transmitted infections, with the lungs being the most common site of primary infection (4, 6). This type of bacteria can invade broken skin causing a suppurative skin infection, which can lead to a pulmonary infection when it enters the respiratory tract (2). In addition, it can cause disseminated infection of the kidneys or other organs through hematogenous dissemination, leading to acute or chronic suppurative granulomatous disease (7). According to statistics, the mortality rate of pulmonary *Nocardia* infection is about 18% to 30%, which is higher in disseminated infections, and can reach 50% after involvement of the central nervous system (8). *Nocardia* is easily misdiagnosed, leading to ineffective treatment. The main reasons include slow growth of *Nocardia*, requiring longer cultrue time, and some strains forming colonies only after 2 weeks; High technical requirements for rapid detection of *Nocardia* and its sensitivity to interference from other microorganisms; Non-specific clinical and imaging features that easily be confused with other bacterial diseases such as pulmonary tuberculosis and cutaneous tuberculosis. These factors may be the important causes of high mortality rates in patients (1, 4, 9). In recent years, due to the rapid development of detection technologies such as mass spectrometry and metagenome next-generation sequencing (mNGS), which have been increasingly used to detect *N. otitidiscaviarum* and other rare taxa, the mortality rate of *Nocardia* infections has decreased (10, 11).

The reason why *Nocardia* is more common in immunocompromised populations may be related to its pathogenic characteristics, as it has strong pathogenicity and can invade the human body through damaged skin, the respiratory tract, the digestive tract (12, 13). In addition, immunocompromised patients have decreased immune system function and weakened immune response ability, making it difficult to effectively resist the invasion and reproduction of *Nocardia*, which significantly increases the risk of infection (14). Studies have shown that most patients with pulmonary or disseminated *Nocardia* disease have immune dysfunction mainly characterized by cellular immune deficiency, and such patients have a high rate of macrophage dysregulation under the long-term action of various inflammatory factors, which partly leads to *Nocardia* susceptibility (15). Therefore, immunosuppression-related diseases such as HIV infection, cancer, chemotherapy, solid organ transplantation (especially lung transplantation), allogeneic hematopoietic stem cell transplantation patients, diabetes, autoimmune diseases, glucocorticoid use, and other diseases that lead to cellular immune deficiency provide a potential basis for *Nocardia* infection (16).

Despite the increasing recognition of *Nocardia*, infections caused by *N. otitidiscaviarum* are relatively rare compared to other *Nocardia* species, accounting for only 3% to 5% of all reported *Nocardia* infections (3, 4). In a study conducted in the United States, only 2.9% (10/347) of all *Nocardia* infections were caused by *N. otitidiscaviarum* (17), and a similar result was obtained in a study conducted in China, which was 5.9% (26/441) (18). The low infection rate of *N. otitidiscaviarum* may be related to its low pathogenicity, limited distribution in soil, or underreporting of cases (19). In addition, there are some differences in pathogenicity and epidemiology between *N. otitidiscaviarum* and other *Nocardia* species. The former usually infects people with specific underlying diseases or immune impairments, while the latter, such as N. *asteroides* and *N. brasiliensis*, are relatively more common in healthy people but can also occur in patients with underlying diseases or immune impairments. In terms of pathogenicity, *N. otitidiscaviarum* can usually cause pulmonary infections, skin and central nervous system infections, while other *Nocardia* mainly enter the body through the respiratory tract, causing primary purulent pulmonary infections, and in some cases, the infection may also spread to other parts, such as the brain, forming brain abscesses (20, 21).

This study reports a case of N. *otitdiscaviarum* pulmonary infection. The patient had a history of bladder and prostate tumors and chronic bronchiectasis. After receiving hormone therapy for newly diagnosed IgA nephropathy, he was infected with N. *otitdiscaviarum* within a short period of time. Previous studies had shown that corticosteroids and immune agents were considered risk factors for *Nocardia* infection. Steinbrink et al. (22) analyzed clinical data of 112 patients with *Nocardia* infection and found that *Nocardia* infection in immunosuppressed patients was associated with the use of high-dose glucocorticoid therapy and hematopoietic stem cell transplantation therapy. A multicenter case-control study in Europe showed that long-term use of high-dose corticosteroids (>20 mg/day prednisone for at least 1 month) was an independent risk factor for *Nocardia* infection (23). Another retrospective study in Israel confirmed that the use of systemic corticosteroids could increase the risk of *Nocardia* infection, especially in patients with chronic lung disease (24). In addition, research reports on kidney disease patients showed that the median dose of steroids for patients with *Nocardia* infection was 20 mg/day, and the median course of treatment was 4–6 months (25). In this case, the reasons for rapid infection with *N. otitidiscaviarum* after hormone therapy potentially included a history of cancer, chronic bronchiectasis, high initial hormone dose (30 mg/day), and the patient’s interest in planting flowers, which increased the possibility of close contact with *Nocardia* in soil. Under the combined effect of these factors, the risk of *N. otitidiscaviarum* infection in this patient was significantly increased.

Similar to the symptoms of pulmonary infections caused by common bacteria, *Nocardia* pneumonia initially present with fever, cough, expectoration, and chest distress (2). As the condition progresses, lung consolidation, increased pleural effusion, dyspnea, and even respiratory failure may occur. In this case, the patient experienced a similar disease progression. As shown in Figure 2, the pulmonary CT showed that the patient’s pulmonary infection was progressively worsening, with increased lung consolidation and pleural effusion, as well as symptoms of respiratory failure. In some previous studies, more than half of pulmonary *Nocardia* patients had extrapulmonary dissemination, with the brain being the most common site of dissemination (7, 8). Therefore, it is recommended that all patients with *Nocardia* pneumonia or disseminated infection undergo cranial imaging examination to rule out intracranial infection. Although the patient in this case experienced mild headaches, listlessness, and slow reaction for several days during the illness, no imaging evidence of intracranial infection was found in the brain MRI examination. However, as shown in Figure 3F, the patient developed severe tongue and lip ulcers in the early stages of the disease. As mentioned earlier, *Nocardia* can cause skin infections, with *N. brasiliensis* being the most common, and the pathogenic factors of *Nocardia* skin infections are mostly horticultural work and trauma (2). Skin *Nocardia* disease needs to distinguish between primary skin *Nocardia* infection and other organ *Nocardia* infections affecting the skin. The former can manifest as skin lymphadenopathy, foot mycosis, and cellulitis, with a few reports of *Nocardia* causing keratitis, bone and joint infections (26). The latter is a manifestation of *Nocardia* infection in other organs accompanied by skin damage. In this case, the patient’s lip and tongue ulcers appeared in the early stages of *N. otitidiscaviarum* infection and gradually improved after the pneumonia was controlled, and no other skin lesions were found in other parts. Therefore, it could be considered that the mucosal lesions of the patient might be related to pulmonary infection caused by *N. otitidiscaviarum*. This phenomenon suggested that skin and mucosal damage may occur earlier than respiratory symptoms and pulmonary imaging changes after *N. otitidiscaviarum* infection, which had not been previously reported.

In this case, the patient was ultimately diagnosed with an *N. otitidiscaviarum* infection, but it was not promptly identified in the early stages of the disease. The reasons for delayed diagnosis might mainly included atypical early clinical symptoms, difficulties in *Nocardia* culture, and drug interference. The gold standard for diagnosing *Nocardia* infection is to isolate and culture *Nocardia* (4). However, we did not find it in the four sputum cultures where samples were taken separately in the early stages of the disease. As mentioned above, some strains of *Nocardia* grow slowly in culture medium, many microbiology laboratories culture sputum at regular times and discard the culture medium too early, resulting in belated diagnosis for some *Nocardia* patients, which might be one of the reasons for early misdiagnosis in this case. In addition, drug interference also needs to be taken into account in this case. The patient was treated with hormone therapy for IgA nephropathy while a low-dose of TMP-SMZ was used to prevent potential infections. Theoretically, a low-dose of TMP-SMZ can reduce the probability of infection in high-risk patients, but some previous clinical studies indicated that a low-dose of TMP-SMZ could not prevent potential infections and might increase the risk of drug resistance (27). According to some treatment guidelines, TMP-SMZ is also one of the preferred medication for treating *Nocardia* infections (5, 28). Therefore, when this patient was administered a low-dose of TMP-SMZ to prevent potential infection, it might also inhibit the growth of *Nocardia*, thereby interfering with the detection of *Nocardia* in sputum. Fortunately, *Nocardia* was found in higher quality BALF specimens and further confirmed as *N. otitidiscaviarum* by mass spectrometry, providing favorable evidence for the diagnosis of the case.

*Nocardia* varies in regional distribution and drug resistance, and treatment recommendations for *Nocardia* may vary from region to region. In the treatment guidelines of the American Society of Transplantation for *Nocardia* infection, TMP-SMZ is recommended as the first choice for transplant patients with mild to moderate pulmonary infection, followed by imipenem combined with amikacin, ceftriaxone, minocycline, or linezolid, with a course of treatment of 6–12 months. For patients with severe pulmonary infection, brain abscesses and disseminated infections, the guidelines recommend imipenem combined with amikacin or TMP-SMZ as the first choice, with a course of treatment of 6–12 months (5). The treatment guidelines for *Nocardia* released by Australia recommend the use of TMP-SMZ for patients with mild to moderate infections, for patients with severe infections, it is recommended to use TMP-SMZ in combination with linezolid, acaricin, imipenem, or meropenem (28). Comparing the guidelines of the United States and Australia, it can be found that the first recommended combination therapy for pulmonary or disseminated infections in the United States is TMP-SMZ combined with imipenem. Although some reports indicated that the resistance rate of *Nocardia* to TMP-SMZ reached 42% and to imipenem ranged from 30 to 55% (28, 29), there were also studies suggesting that early and sufficient drug treatment could still achieve good curative effect (18, 30). In this case, when *Nocardia* was identified in the BALF, TMP-SMZ combined with imipenem were administered immediately according to the guidelines, but it was ineffective and the patient’s condition did not improve. Finally, the pathogen was identified as *N. otitidiscaviarum* by mass spectrometry, and according to the results of drug sensitivity tests, the antibiotic was changed to TMP-SMZ combined with linezolid, after which the patient’s condition improved rapidly. After discharge, the patient received oral maintenance treatment with linezolid and his condition remained stable. Although imipenem is one of the recommended drugs for the treatment of *Nocardia*, the resistance rate in *Nocardia* is increasing, while the resistance rate to linezolid is relatively low. This case further confirmed this, which was consistent with some recent reports (31–33). This case reminded us that early identification of pathogens and personalized treatment based on drug sensitivity results are key to curing patients with *Nocardia* disease. In addition, previous studies suggested that reducing hormone dosage in the short term might help with the treatment of *Nocardia* infection (34). In this case, while receiving antibiotic treatment, the dosage of hormone was gradually decreased, and the patient’s condition gradually improved, indicating that the adjustment of the dosage of hormone might also be related to the rapid improvement of *N. otitidiscaviarum* pneumonia.

## Conclusion

In conclusion, this case reports a patient with a history of cancer and chronic bronchiectasis who was infected with the rare *N. otitidiscaviarum* during hormone therapy for IgA nephropathy. The atypical clinical manifestations of *Nocardia* pneumonia and the characteristics of *Nocardia* itself pose a serious challenge to clinical physicians. Currently, there is still a lack of consistent and effective empirical treatment plans for *Nocardia* infection, and its high severity and mortality rates require full attention from clinical physicians. We recommend that for patients with a history of chronic lung disease, when pulmonary infection occurs during hormone or immunosuppressive therapy for kidney disease, the possibility of *Nocardia* infection should be fully considered, and high-quality specimens should be collected as early as possible. Appropriate bacterial culture methods and efficient identification techniques, such as mass spectrometry and mNGS, should be adopted to promptly identify pathogens, and personalized treatment should be developed based on antibiotic sensitivity tests to save patients’ lives.

## Data Availability

The original contributions presented in this study are included in this article/supplementary material, further inquiries can be directed to the corresponding author.
